# Vasculogenic mimicry is associated with trastuzumab resistance of HER2-positive breast cancer

**DOI:** 10.1186/s13058-019-1167-3

**Published:** 2019-08-06

**Authors:** Ami Hori, Masafumi Shimoda, Yasuto Naoi, Naofumi Kagara, Tomonori Tanei, Tomohiro Miyake, Kenzo Shimazu, Seung Jin Kim, Shinzaburo Noguchi

**Affiliations:** Department of Breast and Endocrine Surgery, Osaka University Graduate School of Medicine, 2-2-E10 Yamadaoka, Suita, Osaka 565-0871 Japan

**Keywords:** Breast carcinoma, ERBB2, Salinomycin, Trastuzumab, Vasculogenic mimicry

## Abstract

**Background:**

Trastuzumab is a drug that targets the receptor tyrosine kinase HER2 and is essential for the treatment of HER2-positive breast cancer. Resistance to the drug leads to severe consequences, including disease recurrence, tumor enlargement, and metastasis. We hypothesized that trastuzumab treatment might be associated with phenotypic switching in HER2-positive breast cancer cells (BCCs), enabling them to escape and survive the effect of trastuzumab.

**Methods:**

We conducted comprehensive immunophenotyping to detect phenotypic changes in HER2-positive BCCs treated with trastuzumab, based on criteria determined a priori. Based on immunophenotyping results, we characterized the vascular phenotypes of HER2-positive BCCs by western blotting, real-time RT-PCR, and tube formation assay. The vascular phenotype of tumor cells from clinical samples was evaluated by staining with periodic acid-Schiff and an anti-CD31 antibody. We explored small molecule inhibitors that suppress tube formation and determined the inhibitory mechanism.

**Results:**

Out of 242 cell surface antigens, 9 antigens were significantly upregulated and 3 were significantly downregulated by trastuzumab treatment. All upregulated antigens were related to endothelial and stem cell phenotypes, suggesting that trastuzumab treatment might be correlated to switching to a vascular phenotype, namely, vasculogenic mimicry (VM). Several VM markers were upregulated in trastuzumab-treated cells, but these cells did not form tubes on Matrigel, a functional hallmark of VM. Upon analysis of three trastuzumab-resistant HER2-positive cell lines, we found that all three cell lines showed tube formation on Matrigel in the presence of angiogenic growth factors including EGF, FGF2, IGF1, or VEGF. Clinically, VM channels significantly increased in surviving cancer cell clusters of surgically removed tumors pretreated with trastuzumab and chemotherapy compared to both surgically removed tumors without prior systemic treatment and tumors biopsied before presurgical treatment with trastuzumab. Finally, we found that salinomycin completely suppressed VM in all three trastuzumab-resistant cell lines through disruption of actin cytoskeletal integrity.

**Conclusions:**

VM promotes metastasis and worsens patient outcomes. The present study indicates that HER2-positive BCCs can exhibit VM in an angiogenic microenvironment after eventually acquiring trastuzumab resistance. The clinical finding supports this in vitro observation. Thus, targeting VM might provide a therapeutic benefit to patients with HER2-positive breast cancer.

**Electronic supplementary material:**

The online version of this article (10.1186/s13058-019-1167-3) contains supplementary material, which is available to authorized users.

## Background

Breast cancer exhibiting gene amplification and/or overexpression of human epidermal growth factor receptor (HER)-2 is called HER2-positive (HER2+) breast cancer. Patients with HER2+ breast cancer account for 15–20% of all patients with invasive breast cancer [1]. HER2 is a receptor tyrosine kinase that has no specific ligands; the protein is activated when it forms a homodimer or when it heterodimerizes with other ligand-binding HER family members, especially HER3. Upon activation, HER2 can activate downstream signaling pathways including the mitogen-activated protein kinase (MAPK) pathway and the phosphoinositide-3 kinase (PI3K)/AKT pathway, resulting in the promotion of cellular proliferation and survival [2]. Thus, HER2+ breast cancer exhibits a distinct and aggressive clinical presentation compared with estrogen receptor (ER)-positive, HER2-negative breast cancer, which is a major subtype of breast cancer often exhibiting less aggressive behavior [3]. The aggressive phenotypes of HER2+ breast cancer include rapid tumor growth and a high incidence of metastasis to vital organs, such as the liver and the brain. Consequently, the prognosis of patients with HER2+ breast cancer was poor compared with that of patients with HER2-negative breast cancer until trastuzumab (Tzm) was developed for the treatment of this devastating disease [4].

Tzm is a humanized monoclonal antibody that recognizes the extracellular domain of HER2. The function of HER2 is disrupted in multiple ways when it is bound to Tzm, leading to growth arrest and apoptosis of HER2+ breast cancer cells [5]. With its potent effect on HER2+ breast cancer, Tzm has made significant contributions to improving patient prognoses. For early HER2+ breast cancer patients, Tzm prevents up to 40% of recurrence during the 10 years after surgery [6], and for metastatic HER2+ breast cancer patients, first-line use of Tzm concurrently with chemotherapy significantly prolongs progression-free survival by 3 months [7]. However, 26% of early HER2+ breast cancer cases recur within 10 years after surgery, and over 70% of metastatic HER2+ breast cancer cases progress within 1 year despite continuous administration of Tzm [6, 7]. Thus, to further improve the results of treatment for HER2+ breast cancer, the mechanism of Tzm resistance needs to be clarified, and treatments overcoming such resistance need to be developed.

One compelling explanation of the mechanism underlying Tzm resistance is that alternative signaling pathways compensate for or override the blockade of the HER2 signaling pathway by Tzm [8]. These alternative pathways, which involve activation of epidermal growth factor receptor (EGFR), HER3, insulin-like growth factor-1 receptor (IGF1R), and hepatocyte growth factor receptor (MET), may lead to reactivation of the MAPK pathway and the PI3K/AKT pathway. Interestingly, pathways that do not directly reactivate the MAPK pathway and the PI3K/AKT pathway are also associated with Tzm resistance. For example, activation of transforming growth factor (TGF)-β and its receptor induces Tzm resistance mediated by the nuclear translocation of SMAD2/3 molecules through induction of the epithelial-mesenchymal transition (EMT) and/or stemness in cancer cells [9, 10]. Other mechanisms include activation of the erythropoietin receptor and EPH receptor A2 (EPHA2), which are typically involved in hematopoiesis and angiogenesis, respectively, but are not commonly involved in homeostasis of the breast epithelium [11, 12]. These findings may indicate that Tzm resistance is associated with phenotypic switching to mesenchymal cells, cancer stem cells (CSCs), hematopoietic cells and endothelial cells. Recent studies have also indicated that such phenotypic plasticity of cancer cells is one of the causes of the acquisition of aggressiveness, which results in tumor progression and metastasis [13, 14]. In the present study, we examined the effect of Tzm loading on the immunophenotype of HER2+ breast cancer cells to clarify whether HER2+ breast cancer cells undergo phenotypic switching to other cell types upon HER2 blockade by Tzm.

## Methods

### Cell culture and generation of Tzm-resistant cell lines

The SKBR3, BT474, and MDA-MB-361 cell lines were purchased from the American Type Culture Collection. The JIMT-1 cell line was purchased from Deutsche Sammlung von Mikroorganismen und Zellkulturen. All cell lines were derived from HER2+ breast cancer cells. The SKBR3 cell line was maintained in McCoy’s 5A medium (Thermo Fisher Scientific, Waltham, MA, USA) supplemented with 10% fetal bovine serum. The BT474, MDA-MB-361, and JIMT-1 cell lines were maintained in DMEM/F12 (Sigma-Aldrich, St. Louis, MO, USA) supplemented with 10% fetal bovine serum. Human umbilical vein endothelial cells (HUVECs) were purchased from PromoCell (Heidelberg, Germany) and were maintained in Endothelial Cell Growth Medium 2 (PromoCell). For generation of Tzm-resistant cell lines, SKBR3 and BT474 cells were grown in DMEM/F12 supplemented with 5% calf serum (Sigma-Aldrich), 4 μg/mL insulin (Thermo Fisher Scientific), 0.5 μg/mL hydrocortisone (Stem Cell Technologies, Vancouver, Canada), and 1 μg/mL (for SKBR3) or 2 μg/mL (for BT474) Tzm (Herceptin; Chugai Pharmaceutical, Tokyo, Japan) for over 6 months.

### Cell growth and cell death assay

SKBR3 and BT474 cells were seeded in 6-cm dishes and cultured overnight in DMEM/F12 supplemented with 5% calf serum, 4 μg/mL insulin, and 0.5 μg/mL hydrocortisone. The next day, 1 μg/mL (for SKBR3) or 2 μg/mL (for BT474) Tzm or vehicle (phosphate-buffered saline) was added to the medium, and the cells were cultured for 5, 7, 9, or 11 days. The cells were detached from the plates, stained with trypan blue and counted using a hemacytometer. For the cell death assay, detached and attached cells cultured in the presence or absence of Tzm as described above for 3 days (SKBR3) or 7 days (BT474) were collected and stained with trypan blue. Dead cells and live cells were separately counted using a Countess Cell Counter (Life Technologies, Carlsbad, CA, USA).

### Cell proliferation assay

Cells cultured with or without Tzm for 10 days were labeled with 10 μM EdU for 2 h by using a Click-iT EdU Flow Cytometry Assay Kit (Thermo Fisher Scientific) and were analyzed with a FACSCanto II flow cytometer (BD Biosciences, San Jose, CA, USA). Dead cells were eliminated with a LIVE/DEAD Fixable Dead Cell Stain Kit (Thermo Fisher Scientific).

### Tzm sensitivity assay

SKBR3 cells and BT474 cells in maintenance medium were detached from dishes with Accutase (BD Biosciences), immunostained with antibodies conjugated with a fluorochrome, and sorted using a FACSAria II cell sorter (BD Biosciences). The antibodies used are listed in Table 1. The sorted cells were seeded into the wells of 96-well plates and cultured overnight in DMEM/F12 supplemented with 5% calf serum, 4 μg/mL insulin, and 0.5 μg/mL hydrocortisone. The next day, 1 μg/mL (SKBR3) or 2 μg/mL (BT474) Tzm or vehicle was added, and the cells were further cultured for 6 days. Then, the number of cells was counted using an IN Cell Analyzer 6000 (GE Healthcare, Chicago, IL, USA). Alternatively, the viability of Tzm-resistant BT474 cells relative to control cells was estimated by using a Cell Counting Kit-8 (Dojindo, Kumamoto, Japan).

**Table 1:** Lists of antibodies used for flow cytometry and western blotting

Antigen	Company	Clone	Catalog no.	
Antibodies for cell sorting				
CD44	BD Biosciences	G44-26 (C26)	563029	
CD142	BD Biosciences	HTF-1	561713	
CD144	BD Biosciences	55-7H1	561714	
CD146	BD Biosciences	P1H12	561013	
CD171	Thermo Fisher Scientific	eBio5G3 (5G3)	17-1719-41	
CD220	BD Biosciences	3B6/IR	559955	
CD221	BD Biosciences	1H7	560934	
ERBB1	BD Biosciences	EGFR.1	563344	
SSEA-1	BD Biosciences	MC480	560886	
Mouse IgG1 κ	Thermo Fisher Scientific	P3.6.2.8.1	12-4714-42	Isotype Control
Mouse IgG2a κ	BD Biosciences	G155-178	550882	Isotype Control
Mouse IgG2b κ	BD Biosciences	27-35	563025	Isotype Control
Mouse IgM κ	BD Biosciences	G155-228	555584	Isotype Control
Primary antibodies for western blotting				
COX2	Cell Signaling Technology	D5H5	12282	
MMP2	Cell Signaling Technology	D8N9Y	13132	
MMP14	Cell Signaling Technology	D1E4	13130	
p-SMAD2 (S465/467)/SMAD3 (S423/425)	Cell Signaling Technology	D27F4	8828	
SMAD2/3	Cell Signaling Technology	D7G7	8685	
p-EPHA4 (Y596)	Abcam	Polyclonal	ab193214	
EPHA4	Abcam	Polyclonal	ab126169	
p-EPHA2 (S897)	Cell Signaling Technology	D9A1	6347	
EPHA2	Cell Signaling Technology	D4A2	6997	
VEGFR2	Santa Cruz Biotechnology	A-3	sc-6251	
ACTB	Cell Signaling Technology	D6A8	12620	
ERBB1	Cell Signaling Technology	D38B1	4267	
FGFR1	Santa Cruz Biotechnology	M2F12	sc-57132	
FGFR2	Cell Signaling Technology	D4L2V	23328	
IGF1R	Cell Signaling Technology	D23H3	9750	
VEGFR1	Santa Cruz Biotechnology	D-2	sc-271789	
Secondary antibodies for western blotting				
Mouse IgG	Jackson ImmunoResearch		715-036-151	
Rabbit IgG	Cell Signaling Technology		7074	

### Comprehensive immunophenotyping

SKBR3 and BT474 cells were grown in DMEM/F12 supplemented with 5% calf serum, 4 μg/mL insulin, 0.5 μg/mL hydrocortisone, and 1 μg/mL (for SKBR3) or 2 μg/mL (for BT474) Tzm or vehicle for 13 days. The cells were detached from the dishes using Accutase and stained with antibodies provided in the Human Cell Surface Marker Screening Panel (BD Biosciences) according to the manufacturer’s protocol. The expression of each antigen was analyzed with a FACSCanto II flow cytometer and FlowJo software (FlowJo, LLC, Ashland, OR, USA). The median fluorescence intensity (MFI) and percentage of positive cells (Pos) were estimated for each antigen. Before analysis, we established the following criteria for determining which antigens were significantly upregulated or downregulated: (1) |Log_2_[MFI_Tzm_] − Log_2_[MFI_Control_]| ≥ 0.4; (2) |[Pos_Tzm_] − [Pos_Control_]| ≥ 2; and (3) both cell lines exhibited similar changes in the expression of an antigen. All of these criteria had to be satisfied. Heat maps were generated using web-based analysis software at http://www.heatmapper.ca [15].

### Tube formation assay

Cells precultured with 1 μg/mL (SKBR3) or 2 μg/mL (BT474) Tzm or control human IgG for 13 days (for Fig. 3d) or Tzm-resistant cell lines and HUVECs cultured in maintenance medium (for Figs. 5, 7, and 8) were detached from dishes using Accutase. The cells were cultured in the wells of 6-, 24-, or 48-well plates coated with Matrigel (Corning, Corning, NY, USA) in Endothelial Basal Medium-2 (EBM-2; Lonza, Basel, Switzerland) supplemented with all reagents of Microvascular Endothelial Cell Growth Medium-2 SingleQuots Supplements and Growth Factors (Lonza) for up to 72 h. This medium, described after this as complete EBM-2, contained four angiogenic growth factors including epidermal growth factor (EGF), fibroblast growth factor 2 (FGF2), insulin-like growth factor 1 (IGF1), and vascular endothelial growth factor (VEGF). For the experiments in Fig. 5d, the Tzm-resistant cell lines were cultured in the wells of 24-well plates coated with growth factor-reduced Matrigel (Corning) in the presence of single angiogenic growth factors or all four growth factors. For the inhibitor experiments appearing in Figs. 7 and 8, the Tzm-resistant cell lines and HUVECs were pretreated with various inhibitors for 2 h. The pretreated cells were cultured in Matrigel-coated wells in complete EBM-2 medium with the same inhibitor for up to 72 h. For Fig. 8h and i, 1 μg/mL Rho Activator II (Cytoskeleton, Inc., Denver, CO, USA) was added 1 h prior to the addition of 0.3 μM salinomycin (Sigma-Aldrich) and continuously supplemented into the medium. Rho Activator II was designed based on the catalytic domain of bacterial cytotoxic necrotizing factors with modifications to increase cell permeability [16, 17]. At the end of the assay, photographs were obtained using a Leica DMi1 phase-contrast microscope with a × 5 objective lens (Leica Microsystems, Wetzlar, Germany). Color images were converted to grayscale, image sizes were reduced, and images were sharpened once for clarity using ImageJ software (National Institutes of Health, Bethesda, MD, USA). Tube formation was quantified by counting the number of tubes formed. When counting, unprocessed photographs were used. A tube was defined as a linear sequence of cells linking two nodes.

### Western blotting

For the data in Fig. 3b, cells cultured in the presence of 1 μg/mL (SKBR3) or 2 μg/mL (BT474) Tzm or control human IgG (Thermo Fisher Scientific) for 13 days were lysed with RIPA buffer supplemented with protease inhibitors and phosphatase inhibitors. For the data in Fig. 5c, cells cultured on Matrigel in complete EBM-2 medium overnight were collected with Corning Cell Recovery Solution and lysed with the same RIPA buffer as above. Protein (5 μg) was loaded into gels for SDS-PAGE. The primary and secondary antibodies used are shown in Table 1. The detailed protocol was described previously [9].

### Quantitative reverse transcription PCR (qRT-PCR)

Cells cultured in the presence of 1 μg/mL (SKBR3) or 2 μg/mL (BT474) Tzm or control human IgG for 13 days were lysed with TRIzol reagent (Thermo Fisher Scientific), and the lysates were subjected to RNA extraction. qRT-PCR was performed using gene-specific TaqMan probes (Thermo Fisher Scientific). The probe IDs were Hs00900055_m1 for VEGFA, Hs00153153_m1 for HIF1A, Hs01026149_m1 for HIF2A, Hs01675818_s1 for TWIST1, and Hs02758991_g1 for GAPDH. The detailed protocol was described previously [9].

### Histological evaluation

A cohort of all HER2+ breast cancer patients who primarily underwent surgery between January 2016 and August 2017 (*N* = 25) and all HER2+ breast cancer patients intending to receive neoadjuvant chemotherapy (NAC; 12 weekly cycles of 80 mg/m^2^ paclitaxel followed by 4 triweekly cycles of 500 mg/m^2^ 5-fluorouracil, 75 mg/m^2^ epirubicin, and 500 mg/m^2^ cyclophosphamide [FEC]) at our institution (*N* = 110) were initially targeted for retrospective analysis. Patients having histories of any preceding malignancies were excluded. The first administration of paclitaxel to the NAC groups with and without Tzm occurred between May 2008 and July 2017. Of the 110 HER2+ breast cancer patients, 27 patients received NAC without Tzm and 78 patients received NAC with Tzm (the first dose of 4 mg/kg followed by 2 mg/kg doses in 12 cycles) concurrently with paclitaxel. In patients who had received complete doses of the planned drugs, we analyzed specimens that contained at least several clusters of invasive cancer cells, in order to observe if VM was present in this remaining cluster of cancer cells, which were considered to be Tzm-resistant. A schematic of the process of patient selection is shown in Fig. 6a. Pathologists at our institution independently determined the extent of remaining cancer cell clusters according to criteria described elsewhere [18], and we analyzed cases of breast cancer for which the postchemotherapeutic effect was estimated as grade 0, 1a, 1b, or 2a. To compare the number of VM channels in NAC-pretreated tumors and untreated tumors, we further chose cases with available untreated tumor samples that were biopsied before NAC (Fig. 6a). Prior to NAC, we performed breast tumor biopsy by using a vacuum-assisted biopsy system (Mammotome; Devicor Medical, Tokyo, Japan) with an 8-gauge biopsy needle. Tumor biopsy samples obtained before NAC were unavailable (2 cases in the NAC without Tzm group and 4 cases in the NAC with Tzm group) in cases where patients were subjected to a biopsy elsewhere before the first visit to our institution. For immunohistochemical (IHC) CD31 and periodic acid-Schiff (PAS) staining, 4-μm-thick slices from formalin-fixed, paraffin-embedded tumor blocks prepared from surgically removed tumors and biopsied tumor samples were deparaffinized and subjected to heat-induced antigen retrieval using Target Retrieval Solution, pH 9.0 (Dako, Santa Clara, CA, USA). The specimens were immunostained with mouse anti-CD31 antibody (JC70A; Dako) and visualized with 3,3′-diaminobenzidine (DAB; Wako, Tokyo, Japan). PAS staining was performed using a Periodic Acid Schiff Stain Kit (ScyTek Laboratories, West Logan, UT, USA) according to the manufacturer’s instructions. After dehydration, clearing, and mounting by standard methods, the specimens were observed using a BX51 light microscope (Olympus, Tokyo, Japan). Clinicopathological evaluation was performed as described previously [9]. This study was approved by the institutional review board of Osaka University Hospital, and written informed consent was obtained from each patient.

### Time-lapse microscopy

Cells were precultured in maintenance medium supplemented with 0 or 4 μM salinomycin for 2 h. Then, the cells were collected using Accutase and seeded into 35-mm dishes coated with Matrigel. The cells were cultured in complete EBM-2 medium with 0 or 4 μM salinomycin under an IX83 inverted microscope (Olympus) equipped with an incubator at 37 °C in 5% CO_2_/95% air. Phase-contrast images were acquired beginning 15 min after seeding at time intervals of 2 min 30 s up to 14 h.

### Actin fiber staining and confocal microscopy

Tzm-resistant SKBR3 cells were seeded and incubated on Matrigel-coated 4-well chamber slides (Thermo Fisher Scientific) in complete EBM-2 medium for 30 min. Then, the medium was replaced with Hank’s balanced salt solution supplemented with 0 or 4 μM salinomycin, and the cells were further incubated for 2 h. The cells were fixed with 4% paraformaldehyde for 10 min at room temperature. After permeabilization with 0.2% Triton X-100 for 2 min, filamentous actin (F-actin) was stained with ActinGreen 488 Ready Probe (Thermo Fisher Scientific) for 30 min. Nuclei were counterstained with DAPI, and confocal images were obtained using an FV10i confocal laser scanning microscope (Olympus). The amount of F-actin in a cell was quantified using ImageJ software and was represented as integrated density.

### Cell migration assay

Cells were seeded into a 35-mm μ-Dish with a 2-well culture insert (Ibidi, Martinsried, Germany) and cultured overnight in complete EBM-2 medium. The next day, DMSO or 1 μM salinomycin was added to the medium, and the cells were cultured for another 2 h. For the data in Fig. 8g, 2 μg/mL Rho Activator II was added 30 min prior to the addition of 0.5 μM salinomycin. Then, the inserts were removed, and phase-contrast images were obtained several times during a period of up to 36 h using a Leica DMi1 phase-contrast microscope with a × 5 objective lens.

### Rho-GTP pulldown assay

JIMT-1 cells were cultured on Matrigel in complete EBM-2 medium. After the medium was replaced by Hank’s balanced salt solution with DMSO or 0.5 μM salinomycin, the cells were cultured for another 2 h. Occasionally, 2 μg/mL Rho Activator II was added 30 min prior to the addition of 0.5 μM salinomycin. Cell lysates were prepared and subjected to GTP-bound Rho pulldown assays using an Active Rho Detection Kit (Cell Signaling Technology, Danvers, MA, USA) under the manufacturer’s instructions. RhoA was detected using rabbit anti-RhoA antibody (Cell Signaling Technology, #2117).

### Statistics

Statistical analysis was performed using GraphPad Prism 6 (GraphPad Software, Inc., San Diego, CA, USA) and SPSS software (IBM, Armonk, NY, USA). For parametric analysis, Student’s *t* test was applied unless otherwise specified. For nonparametric analysis, Mann-Whitney *U* test was applied. Dunn’s multiple comparison test was performed for the data shown in Fig. 6d. For the pairwise comparisons in Fig. 6e, the Wilcoxon matched-pairs signed-rank test was used. For contingency analysis, the chi-square test was applied. A *P* value of less than 0.05 was considered significant. All tests were described as two-tailed.

## Results

### Comprehensive immunophenotyping of Tzm-loaded HER2+ breast cancer cells

**Fig. 1:**
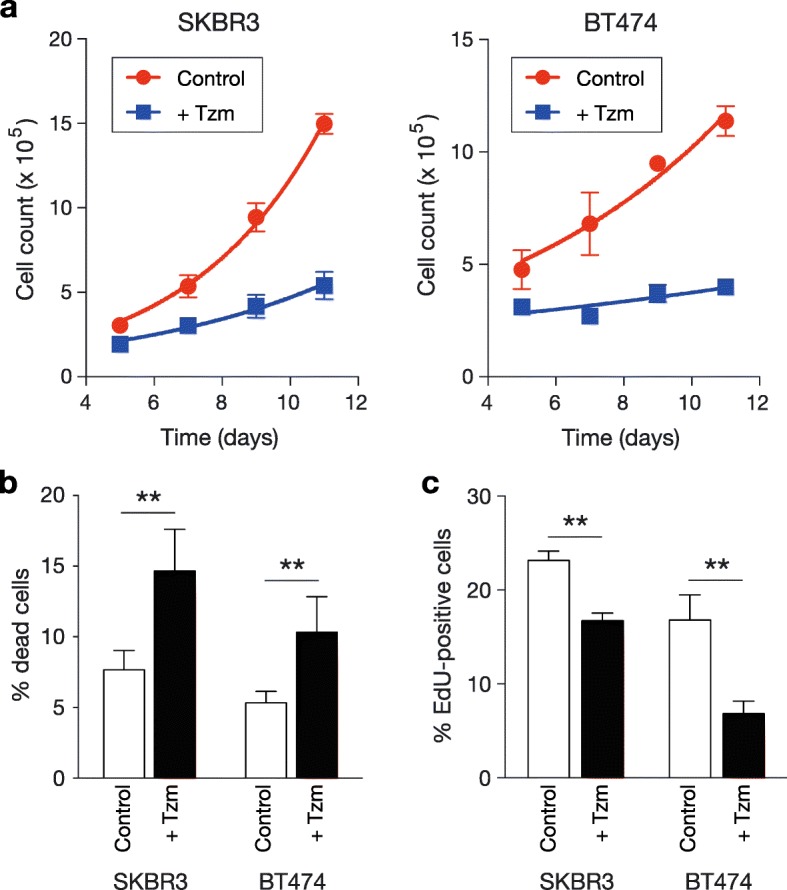
Effect of short-term Tzm loading on the growth of HER2+ breast cancer cell lines. **a** Cell growth assay. Growth curves of SKBR3 and BT474 cells cultured in the presence or absence of Tzm are shown (*n* = 2; mean ± SD). **b** Cell death assay. The percentages of dead cells among cells cultured in the presence or absence of Tzm for 3 days (SKBR3) or 7 days (BT474) are shown (*n* = 6; mean ± SD). **P < 0.01. **c** Cell proliferation assay. The percentages of 5-ethynyl-2′-deoxyuridine (EdU)-positive cells cultured with or without Tzm for 7 days are shown (*n* = 3; mean ± SD). **P < 0.01

Initially, we assumed that a phenotypic change as a result of short-term loading of Tzm might represent a primary change, although it might be subtle; on the other hand, phenotypic changes associated with long-term loading of Tzm might include many easily detectable secondary changes that mask a significant primary change. Thus, we sought to compare HER2+ breast cancer cell lines cultured with Tzm for a short period (13 days) with those cultured without Tzm. We used two HER2+ cell lines, SKBR3 and BT474, which are estrogen receptor (ER)-negative and ER-positive, respectively. The optimal Tzm concentrations in the culture system were determined to be 1 μg/mL for SKBR3 and 2 μg/mL for BT474; these concentrations were the minimum concentrations producing a maximum effect in each dose-response curve [9] and were used to prevent overloading of Tzm. First, we clarified the effect of short-term Tzm loading on the growth of HER2+ breast cancer cell lines. During short-term culture with Tzm, the growth of both cell lines was suppressed (Fig. 1a). This suppressed growth was due to both induction of cell death and reductions in cellular proliferation (Fig. 1b, c). Although statistically significant, the differences in cell death and proliferation between the two groups were so small that cells surviving Tzm loading for 13 days were considered not to have represented a minor fraction of the original population for either group.

**Fig. 2:**
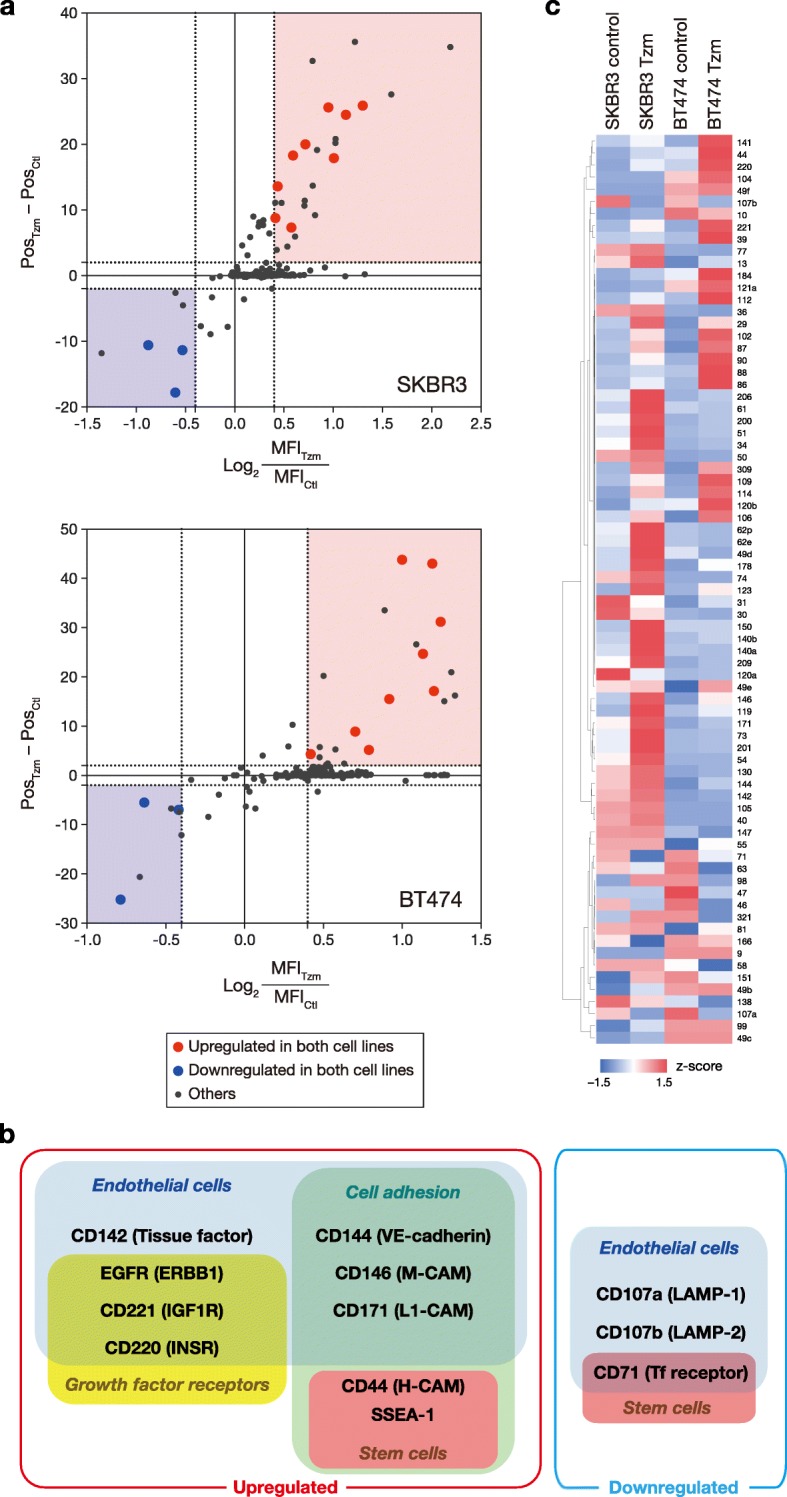
Comprehensive immunophenotyping of HER2+ breast cancer cell lines cultured with or without Tzm for 13 days. **a** Differences in the logarithms of MFI (*x*-axis) and Pos (*y*-axis) of 242 cell surface antigens on SKBR3 and BT474 cells. The light red and light blue areas denote predefined areas of significantly upregulated and downregulated antigen expression, respectively. **b** Functional classification of significantly upregulated or downregulated cell surface antigens. **c** Heat map of the positivity rates of cell surface antigens commonly expressed in endothelial cells. The number in each line represents the cluster of differentiation (CD) number

To detect the phenotypic changes in HER2+ breast cancer cells loaded with Tzm, we conducted comprehensive immunophenotyping of Tzm-loaded cells and control cells using a BD Lyoplate Human Cell Surface Marker Screening Panel. We used this method because the detection of marker expression with flow cytometry is sensitive and quantitative. We evaluated the MFI and Pos values for 242 cell surface markers in Tzm-loaded cells and control cells (Fig. 2a and Additional file 1), and the results indicated that the expression of most, though not all, cell surface markers in Tzm-loaded cells did not substantially differ from that in control cells. The criteria we had determined a priori identified 9 antigens as significantly upregulated and 3 antigens as significantly downregulated by Tzm loading (Fig. 2b). Notably, 7 upregulated antigens, including CD144/vascular endothelial-cadherin (VE-cadherin), were endothelial cell markers, and 2 upregulated antigens, including CD44 and SSEA-1, were stemness markers. The heat map of 75 cell surface antigens expressed in endothelial cells among the 242 antigens evaluated (as determined by the Human Cell Differentiation Molecules organization) reveals that most of the antigens were upregulated in Tzm-loaded cells compared with their control counterparts (Fig. 2c). These findings allow us to hypothesize that Tzm loading might be related to vasculogenic mimicry (VM); VM is an example of tumor cell plasticity in which tumor cells form de novo perfusable vascular-like networks, and genes promoting VM are associated with endothelial, stemness, and hypoxia signaling pathways [19].

### Tzm loading is partially associated with vascular features in HER2+ breast cancer cells

**Fig. 3:**
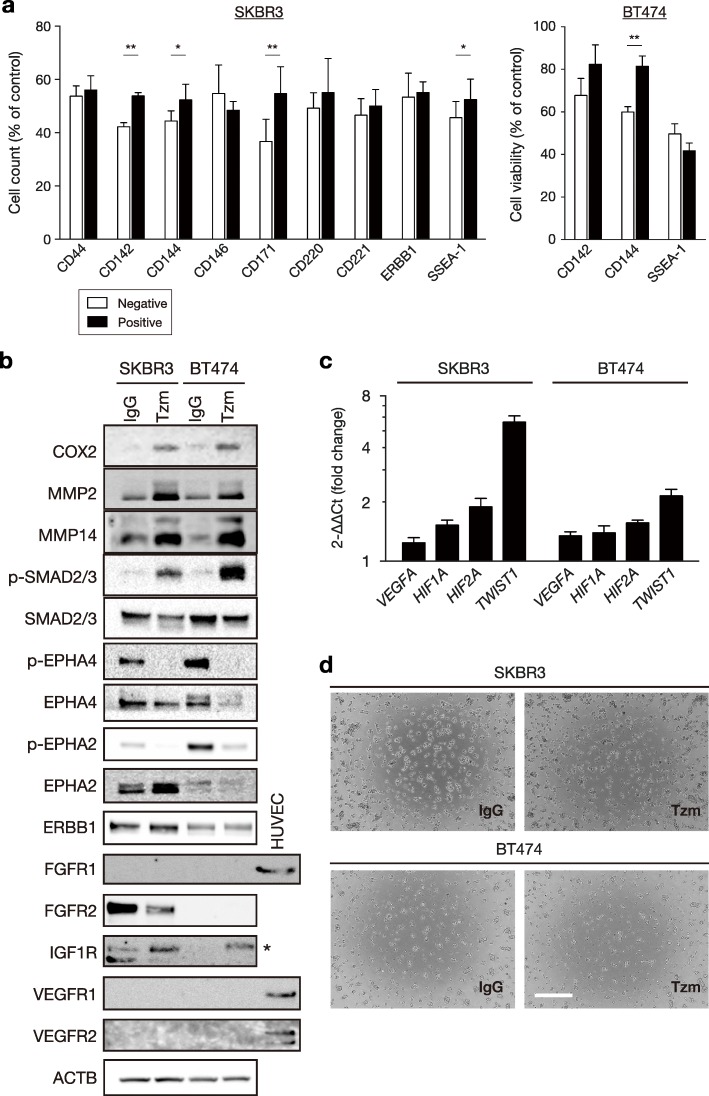
Tzm loading partially induced vascular features in HER2+ breast cancer cells. **a** Tzm sensitivity assay. SKBR3 and BT474 cells positive and negative for each cell surface antigen were cultured in the presence or absence of Tzm for 6 days, and cell counts relative to those of Tzm-absent controls are shown (*n* = 4; mean ± SD). *P < 0.05; **P < 0.01. **b** Western blots of VM markers in cells loaded with IgG or Tzm for 13 days. HUVECs served as positive controls for FGFR1, VEGFR1, and VEGFR2 expression. *, IGF1R protein. **c** Fold changes in mRNA expression of VM markers in cells loaded with Tzm for 13 days relative to control cells as determined by qRT-PCR. mRNA expression was determined by the 2−∆∆Ct method using TaqMan probes (*n* = 3; mean ± SD). **d** Tube formation assay using SKBR3 and BT474 cells precultured with IgG or Tzm for 13 days. Representative photographs are shown. Scale bar, 0.5 mm

To determine whether the upregulation of each cell surface marker was associated with Tzm sensitivity, the two cell lines grown in maintenance medium without Tzm loading were FACS-sorted by each marker and were subjected to Tzm sensitivity assays. CD142, CD144, CD171, and SSEA-1 were significantly associated with Tzm resistance in SKBR3 cells, and CD144 was significantly associated with Tzm resistance in BT474 cells (Fig. 3a).

Next, we examined the expression of various VM markers in Tzm-loaded cells. As expected, COX2, MMP2, MMP14, and phosphorylated SMAD2/3 were upregulated in Tzm-loaded cells compared with control cells, as were *VEGFA*, *HIF1A*, *HIF2A*, and *TWIST1* mRNA (Fig. 3b, c). We then performed a tube formation assay to examine whether cells formed tube-like structures on Matrigel, which is considered a hallmark process of VM. Tzm-loaded SKBR3 cells and BT474 cells did not exhibit tube formation on Matrigel in the presence of four angiogenic growth factors, including VEGF, IGF1, FGF2, and EGF (Fig. 3d). To determine why tubes did not form, we explored the expression of several angiogenic receptors: phosphorylation of both EPH receptor A2 and its functional counterpart (EPH receptor A4) was suppressed, presumably because phosphorylation of EPH receptors mediated by the HER2 tyrosine kinase was inhibited by Tzm [20]. Furthermore, the expressions of FGFR1, VEGFR1, and VEGFR2, all of which play a pivotal role in VM, were not observed in any cells (Fig. 3b). These results indicate that short-term Tzm loading is partially associated with vascular phenotypes in HER2+ breast cancer cells.

### Tzm-resistant cells exhibit VM

**Fig. 4:**
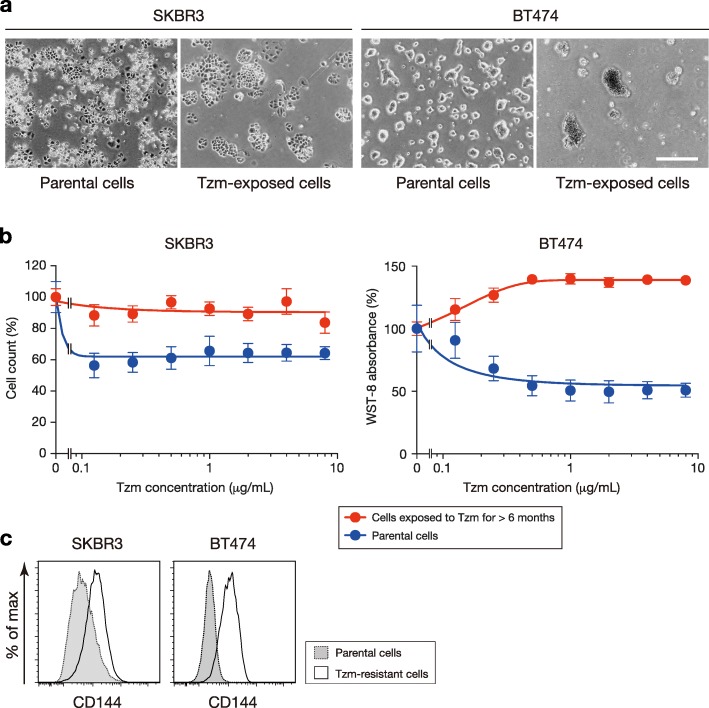
Generation of Tzm-resistant HER2+ breast cancer cells. **a** Morphology of parental cells and cells treated with Tzm for more than 6 months. Note that the BT474 cells loaded with Tzm formed clusters that were not adherent to culture vessels. Scale bar, 0.5 mm. **b** Tzm sensitivity assay. Dose-response curves of SKBR3 cells and BT474 cells (*n* = 4; mean ± SD) are shown. **c** Expression of CD144 (VE-cadherin) in Tzm-resistant SKBR3 cells and BT474 cells compared with parental cells

**Fig. 5:**
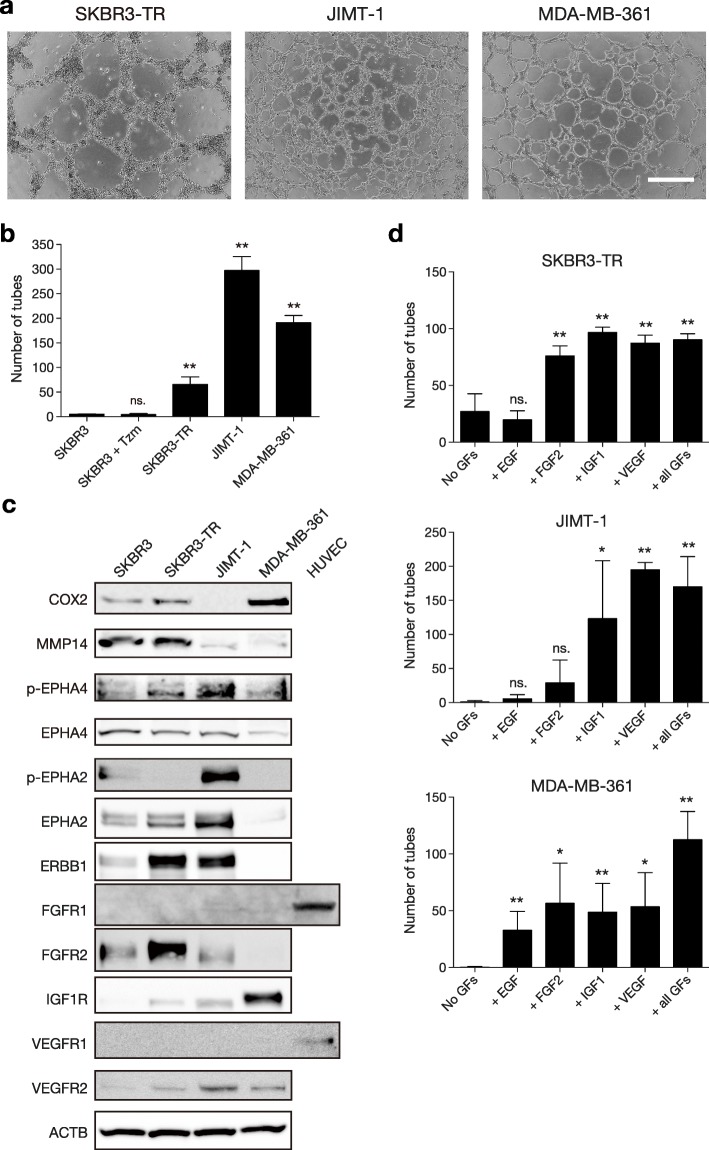
Tzm-resistant HER2+ breast cancer cells exhibited VM. **a** Tube formation assay using three Tzm-resistant cell lines. Representative photographs are shown. Scale bar, 0.5 mm. **b** The numbers of tubes formed are shown (*n* = 4; mean ± SD). ns., not significant; **P < 0.01. **c** Western blots of various VM markers and angiogenic growth factor receptors in Tzm-resistant cells cultured overnight on Matrigel in the medium used for the tube formation assay. HUVECs served as positive controls for FGFR1 and VEGFR1. **d** The effect of each angiogenic growth factor (GF), including EGF, FGF2, IGF1, and VEGF, on tube formation. The numbers of tubes formed are shown (*n* = 4; mean ± SD). ns., not significant; *P < 0.05; **P < 0.01

Taking the results described above into account, we assumed that long-term Tzm loading, which gives rise to Tzm resistance, might be associated with a complete VM phenotype in HER2+ breast cancer cells. We cultured SKBR3 cells and BT474 cells in the presence of Tzm for more than 6 months. BT474 cells loaded with Tzm became completely unattached to culture vessels and grew to form spheroid-like structures, while SKBR3 cells loaded with Tzm remained adherent (Fig. 4a). Both cell lines acquired resistance to Tzm (Fig. 4b). The expression of CD144 in both Tzm-resistant cell lines was upregulated compared with that in the parental cell lines, confirming the relevance of this molecule to Tzm resistance (Fig. 4c). We did not use Tzm-resistant BT474 cells for further experiments because the cells were not adherent to any surfaces; instead, we used two Tzm-resistant HER2+ cell lines, JIMT-1 and MDA-MB-361, in addition to Tzm-resistant SKBR3 cells (designated SKBR3-TR). The JIMT-1 cell line is derived from a patient who received Tzm treatment [21], and the MDA-MB-361 cell line is primarily resistant to Tzm [22]. When these cell lines were subjected to tube formation assays performed in the presence of VEGF, IGF1, FGF2, and EGF, they all exhibited tube formation (Fig. 5a, b).

To elucidate the molecular basis of VM in these cell lines, the expression of VM markers and growth factor receptors involved in angiogenesis, including ERBB1, FGFR1/2, IGF1R, and VEGFR1/2, was examined. Multiple angiogenic growth factor receptors as well as several VM markers were expressed in these cell lines, though the extent and pattern of expression were different among the cell lines (Fig. 5c). Notably, VEGFR2, which plays a central role in VM, was present in all the cell lines. To examine the effect of each angiogenic growth factor on VM in these cell lines, we subjected the cells to tube formation assays in the presence of one of four growth factors including VEGF, IGF1, FGF2, and EGF. VEGF and IGF1 promoted tube formation in all the cell lines, while FGF2 and EGF promoted tube formation in one or two cell lines (Fig. 5d). These results indicate that Tzm-resistant HER2+ breast cancer cells that express angiogenic growth factor receptors exhibit VM in response to multiple angiogenic growth factors. Thus, one can assume that a drug targeting an angiogenic growth factor or its receptor may not effectively inhibit VM, because other growth factor pathways can quickly compensate for the blocked VM pathways.

### Clinical implications of VM in HER2+ breast cancer

**Fig. 6:**
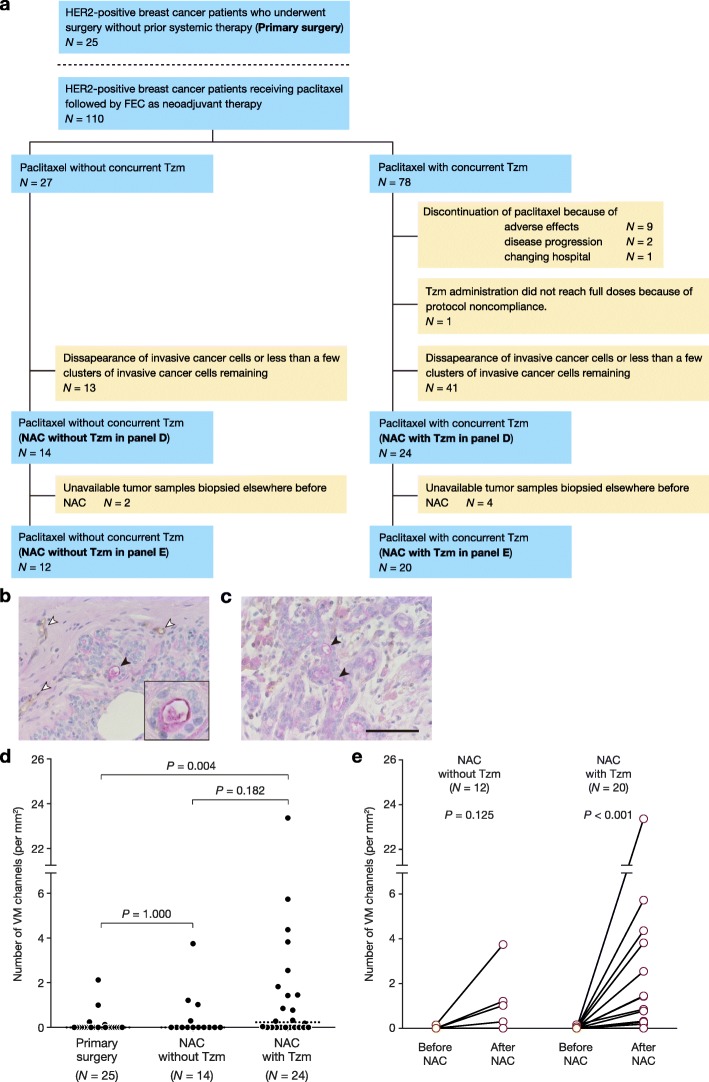
Clinical implications of VM in HER2+ breast cancer. **a** Diagram showing the process of selection of HER2+ breast cancer patients. Beige colored boxes represent excluded patients. **b**, **c** Examples of VM channels consisting of CD31-negative PAS-positive cells (black arrowheads) in HER2+ breast tumors. The white arrowheads denote CD31-positive PAS-positive capillaries. Inset (**b**): a magnified image of a VM channel. Scale bar, 100 μm. **d** The number of VM channels observed in a section of surgically removed tumors. *P* values were calculated by Dunn’s multiple comparison test. Broken lines depict median values. **e** Comparison of the number of VM channels present in tumors obtained before and after neoadjuvant chemotherapy (NAC) in the NAC without Tzm group (left) and the NAC with Tzm group (right). *P* values were calculated by the Wilcoxon matched-pairs signed-rank test

The results of our in vitro study indicate that VM is associated with Tzm resistance. To validate this finding in breast cancer patients, we examined VM in tumor specimens surgically removed from HER2+ breast cancer patients, who had received or not received NAC with or without Tzm. The detailed selection process for patients receiving NAC is described in Fig. 6a. Twenty-five patients who had not received neoadjuvant therapy (primary surgery group), 14 patients who had received neoadjuvant paclitaxel without Tzm (NAC without Tzm group), and 24 patients who had received neoadjuvant paclitaxel with concurrent Tzm (NAC with Tzm group) were analyzed. Menopausal status, tumor size, and lymph node metastasis were statistically different among the three groups (Table 2), suggesting that NAC was chosen more frequently as a primary therapy for younger women with more advanced breast cancer. We recognized VM in tumor tissue as a channel consisting of PAS-positive, CD31-negative cells [23] (Fig. 6b, c). Number of VM channels per unit area was significantly larger in surgically removed tumors of the NAC with Tzm group than that in surgically removed tumors of the primary surgery group (*P* = 0.004; Fig. 6d). For paired tumor samples obtained before and after NAC, the number of VM channels was significantly increased after NAC in the NAC with Tzm group, while the difference in the number of VM channels was not significant in the NAC without Tzm group (Fig. 6e). These results indicate that the in vitro findings are also applicable to HER2+ breast cancer patient samples.

**Table 2:** Association of primary therapy with clinicopathological factors

	Primary surgery *N* = 25 (cases)	NAC^*1^ without Tzm *N* = 14 (cases)	NAC with Tzm *N* = 24 (cases)	*P* ^*2^
Menopausal status				0.002
Premenopausal	6	4	17	
Postmenopausal	19	10	7	
Tumor size				0.034
T1 and T2	24	9	20	
T3 and T4	1	5	4	
Lymph node metastasis				0.011
Negative	17	3	9	
Positive	8	11	15	
Estrogen receptor				0.446
Negative	8	4	4	
Positive	17	10	20	
Progesterone receptor				0.345
Negative	14	8	9	
Positive	11	6	15	
Histological grade				0.183
1 and 2	12	10	17	
3	13	4	7	
Histological type				0.510
Invasive ductal carcinoma	22	12	23	
Others	3	2	1	

^*1^Neoadjuvant chemotherapy. ^*2^Chi-square test

### Salinomycin inhibits VM in Tzm-resistant cells through inactivation of Rho-GTPases

**Fig. 7:**
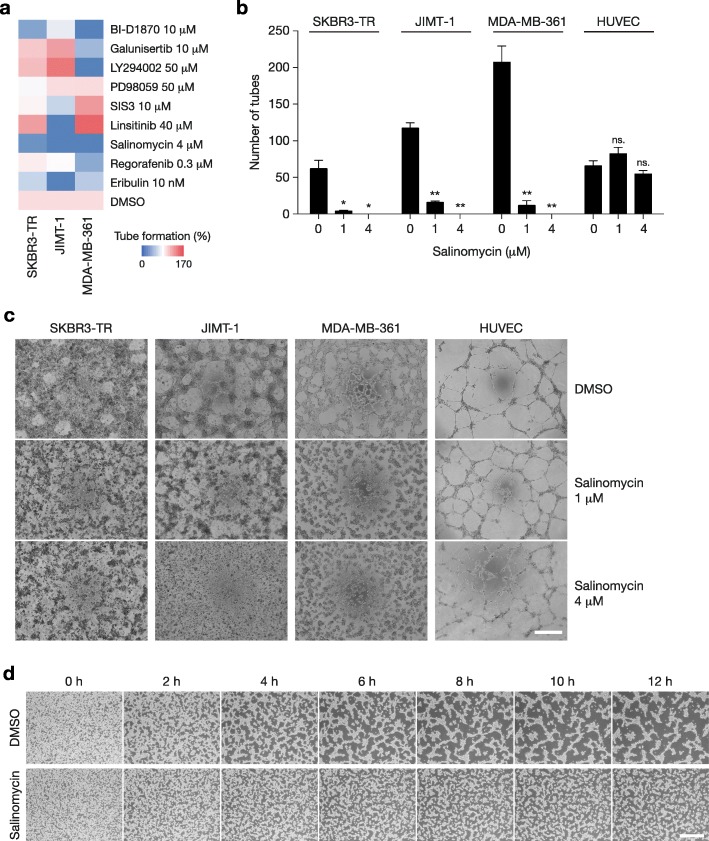
Salinomycin strongly inhibited VM in Tzm-resistant HER2+ breast cancer cells. **a** Percentages of tube formation for three Tzm-resistant cell lines (SKBR3-TR, JIMT-1, and MDA-MB-361) in the presence of 9 small molecule inhibitors compared with that in DMSO controls. The color key indicates the percentage of tube formation. **b** Tube formation assay for Tzm-resistant cell lines and HUVECs cultured on Matrigel in the presence of 0, 1 or 4 μM salinomycin for up to 72 h. The numbers of tubes formed are shown (*n* = 3; mean ± SD). ns., not significant; *P < 0.05; **P < 0.01. **c** Representative photographs showing tube formation in Tzm-resistant cell lines and HUVECs. Scale bar, 0.5 mm. **d** Time-lapse microscopy of tube formation in MDA-MB-361 cells in the presence or absence of 4 μM salinomycin. Scale bar, 0.5 mm


Finally, we searched for a drug that might effectively inhibit VM in Tzm-resistant cells. Among 9 small molecule inhibitors screened, we found that salinomycin, a drug that selectively targets breast CSCs [24], strongly inhibited tube formation in three Tzm-resistant cell lines (Fig. 7a). The validation experiments showed that 4 μM salinomycin completely inhibited tube formation in the three Tzm-resistant cell lines, while the same concentration of salinomycin did not inhibit tube formation in HUVECs (Fig. 7b, c). Time-lapse microscopy of tube formation in MDA-MB-361 cells confirmed the inhibition of tube formation by salinomycin, and it appeared that cell shape and motility were affected (Fig. 7d and Additional files 2 and 3).

**Additional file 2:** Time-lapse video showing a tube formation assay using MDA-MB-361 cells treated with DMSO. (MOV 7111 kb)



**Additional file 3:** Time-lapse video showing a tube formation assay using MDA-MB-361 cells treated with 4 μM salinomycin. (MOV 7291 kb)


**Fig. 8:**
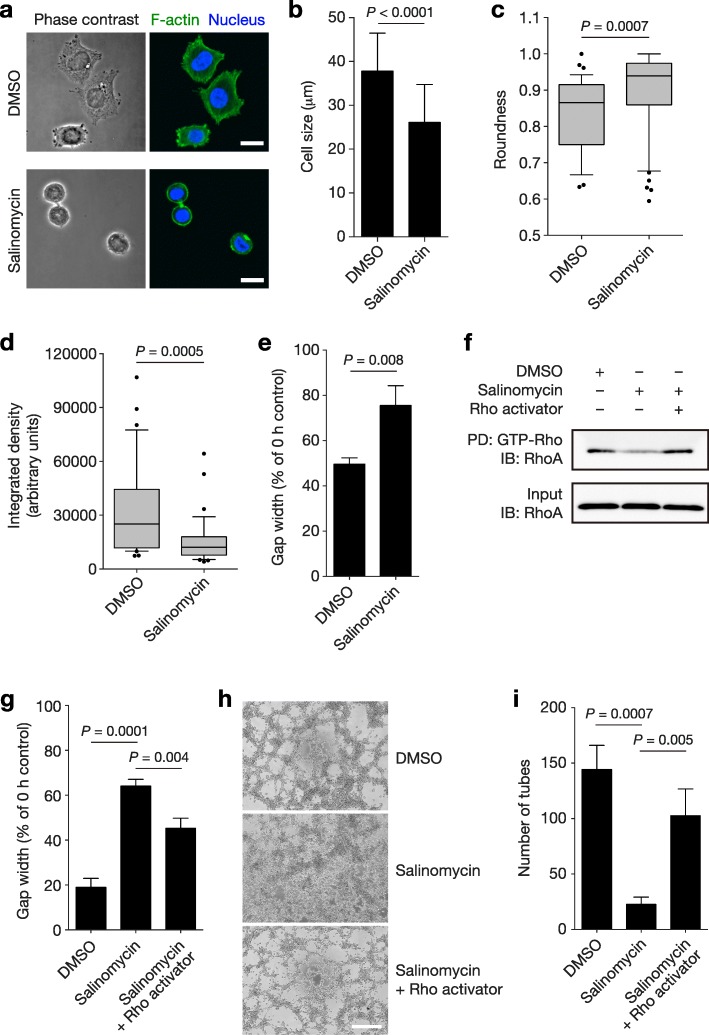
Salinomycin inhibited VM through inhibition of Rho-GTPases. **a** Morphology of SKBR3-TR cells incubated with DMSO or 4 μM salinomycin for 2 h. Representative confocal microscopy photographs of cells stained with Alexa Fluor 488-conjugated phalloidin and DAPI are shown. Scale bar, 20 μm. **b** Sizes of SKBR3-TR cells treated with DMSO (*n* = 37; mean ± SD) or 4 μM salinomycin (*n* = 50; mean ± SD). **c** Box plots of the roundness of SKBR3-TR cells treated with DMSO (*n* = 37) or 4 μM salinomycin (*n* = 50). The whiskers represent the 10th–90th percentiles. *P*, Mann-Whitney U test. **d** Box plots of the integrated density, which represents the amount of F-actin per cell, of SKBR3-TR cells treated with DMSO (*n* = 37) or 4 μM salinomycin (*n* = 50). The whiskers depict the 10th–90th percentiles. *P*, Mann-Whitney U test. **e** Cell migration assay showing the gap width at 36 h (percentage of gap width at 0 h) for SKBR3-TR cells treated with DMSO or 1 μM salinomycin (*n* = 3; mean ± SD). **f** Pulldown assay of GTP-bound Rho-GTPases extracted from JIMT-1 cells treated with DMSO, salinomycin, or salinomycin plus Rho Activator II. PD, pulldown; IB, immunoblot. **g** Cell migration assay using JIMT-1 cells treated with DMSO, 0.5 μM salinomycin or 0.5 μM salinomycin plus 2 μg/mL Rho Activator II (*n* = 3; mean ± SD). **h** Representative photographs showing tube formation in JIMT-1 cells treated for 24 h with DMSO, 0.3 μM salinomycin or 0.3 μM salinomycin plus 1 μg/mL Rho Activator II. Scale bar, 0.5 mm. **i** The numbers of tubes formed are shown (*n* = 3; mean ± SD)

These results prompted us to examine if salinomycin affected cytoskeletal integrity, which might cause the observed changes in cell shape and reductions in cell motility. As expected, SKBR3-TR cells treated with salinomycin showed reduced cell sizes and increased roundness (Fig. 8a–c). Phalloidin staining demonstrated that the amount of F-actin in each cell was decreased, suggesting that salinomycin affected the integrity of the actin cytoskeleton (Fig. 8a, d). Further, salinomycin significantly inhibited cell migration, consistent with the results of time-lapse microscopy (Fig. 8e). We next examined whether the Rho-GTPases, which are master regulators of the actin cytoskeleton, were involved in the inhibition of VM by salinomycin. GTP-bound Rho was reduced in JIMT-1 cells treated with salinomycin but restored by the Rho activator (Fig. 8f), indicating that salinomycin was able to inactivate Rho-GTPases. Accordingly, activation of Rho restored the reduction in cell motility (Fig. 8g) and the inhibition of VM (Fig. 8h, i) caused by salinomycin. These results indicate that salinomycin inhibits VM through inactivation of Rho-GTPases that regulate the integrity of the actin cytoskeleton.

## Discussion

By virtue of its specific effects on cancer cells, molecular targeted therapy has rapidly become prevalent and currently plays a central role in cancer treatment. Tzm is undoubtedly one of the most commonly used molecular targeted drugs in breast cancer as well as in other types of cancers overexpressing HER2. In the present study, comprehensive immunophenotyping used as a minimally biased screening method revealed that Tzm might be associated with a phenotypic switch towards vascular features. The subsequent results indicated that VM was associated with Tzm resistance in HER2-positive breast cancer cells in vitro and in the clinical settings. In terms of the causal relationship between VM and Tzm resistance, it was unlikely that VM would result in Tzm resistance; we examined whether neutralizing anti-CD144 antibodies, such as BV9 and Cad-5 [25], could restore the Tzm sensitivity of CD144-positive SKBR3 and BT474 cells as well as Tzm-resistant SKBR3 and BT474 cell lines. However, both antibodies had no effects on Tzm sensitivity, suggesting that upregulated CD144, which is a key molecule of VM [19], did not confer Tzm resistance. Rather, we hypothesized that in cells predisposed to Tzm resistance and the vascular phenotype, long-term HER2 blockade by Tzm leads to the activation of alternative signaling pathways that contribute to the acquisition of more aggressive phenotypes including drug resistance and VM. Furthermore, we showed that EGFR, FGFR2, IGF1R, and VEFGR2 were upregulated in Tzm-resistant cell lines. Previous studies reporting that BRAF inhibition in melanoma cells induces a proliferative phenotype and metastasis by reactivating the downstream MAPK pathway [26, 27] may also support our hypothesis. The signaling pathways including EGFR, FGFR2, IGF1R, and VEFGR2 have been reported to promote both cell survival and angiogenesis [28–32]; consequence of the activation of these alternative signaling pathways may depend upon the cellular context and the surrounding microenvironment.

The presence of VM has been confirmed in aggressive cancer types such as malignant melanoma and glioblastoma [33–35]. A meta-analysis has revealed that VM is significantly associated with worse outcomes in cancer patients [36]. In breast cancer, approximately 50% of cases classified as the hormone receptor-negative HER2-negative (triple-negative) subtype, considered to be the most aggressive subtype of breast cancer, have been shown to exhibit VM through analysis of clinical samples [37]. In our cohort, compared to that in tumors without prior systemic treatment, VM significantly increased in cancer cell clusters that had survived Tzm-based chemotherapy (14/24 cases; 58%), suggesting that VM is associated with more malignant phenotypes in HER2+ breast cancer.

We showed that Tzm resistance was associated with VM using clinical samples. Specifically, we revealed an increase in VM channels in tumors treated with Tzm-containing chemotherapy using paired tumor samples obtained before and after NAC, which strongly supports our findings. The non-significant differences in the number of VM channels between the NAC with Tzm group and the NAC without Tzm group may have been because of the limited number of patients that were examined. Alternatively, chemotherapeutic drugs may weakly induce VM (Fig. 6e, left), reducing the statistical significance of the difference between the two groups. VM accelerates cancer progression processes, such as tumor growth and metastasis and therefore could be a significant target for cancer treatment [33, 38]. Importantly, cancer cells that are programmed for VM can metastasize to distant organs [39, 40]. Thus, it is likely that VM causes disease progression associated with Tzm resistance. Suppression of VM might prevent metastasis to the liver and brain, both of which are frequent metastatic sites for HER2+ breast cancer [41], and thereby improve the prognosis of patients with advanced and metastatic HER2+ breast cancer. Furthermore, the strategy employed to suppress VM in metastatic HER2+ breast cancer could also be used in the treatment of other aggressive cancer types, such as triple-negative breast cancer.

Our finding that multiple growth factors and their receptors could promote VM in Tzm-resistant cells predicted that an inhibitor targeting a single signaling pathway would have a limited suppressive effect on VM, since other signaling pathways would immediately compensate and eventually restore the process of switching to VM phenotype. In fact, inhibitors such as BI-D1870 (an inhibitor of RSK that activates EPHA2), galunisertib (a TGF-β inhibitor), LY294002 (a PI3K inhibitor), PD98059 (a MAPK inhibitor), SIS3 (a SMAD3 inhibitor), and linsitinib (an IGF1R inhibitor) showed only limited efficacy. Interestingly, regorafenib, a multikinase inhibitor of several angiogenic pathways including the VEGFR pathway, also showed only a limited effect, suggesting that VM does not solely depend upon VEGFR and that pre-existing antiangiogenic drugs may therefore not effectively suppress VM. We found that salinomycin, a potassium ionophore used as an anticoccidial drug, completely suppressed VM. The most prominent property of this drug is that it selectively kills CSCs [24]. Thus, VM and cancer cell stemness are likely to have common vulnerability that can be targeted by salinomycin, as is discussed below. In combination with the finding that Tzm was associated with the expression of several CSC markers including CD44 and SSEA-1, this finding is interesting because recent studies have indicated a relationship between VM and CSCs [34, 35, 42]. Therefore, it is worth investigating whether VM is associated with CSCs because VM may be initiated by CSCs [43].

We demonstrated a detrimental effect of salinomycin on the actin cytoskeleton via inhibition of Rho-GTPase; the same mechanism of action with respect to cell migration has been described previously using pancreatic cancer cells [44]. On the other hand, salinomycin has been reported to be an ionophore that sequesters iron in lysosomes, and the toxicity of iron leads to apoptosis in CSCs [45]. Given these observations, the suppressive effect of salinomycin on Rho-GTPase might be a secondary result. A possible mechanism could be that reactive oxygen species produced due to salinomycin-induced iron accumulation [45, 46] might downregulate the activity of RhoA; this mechanism has been shown in the context of reactive oxygen species, the production of which is mediated by Rac [47]. Our results clearly show that actin cytoskeleton integrity can be a promising target for VM inhibition; however, validation of this finding by performing animal experiments is definitely required, and this strategy of VM inhibition could potentially be further improved by the use of RhoA and actin inhibitors.

## Conclusions

The results of the present study indicate that loss of Tzm sensitivity in HER2+ breast cancer cells leads to VM. Tzm-resistant HER2+ breast cancer cells exhibit VM in an angiogenic microenvironment. Salinomycin effectively suppresses VM by inhibiting actin cytoskeletal integrity. As VM causes disease progression, including metastasis, and worsens patient outcomes, suppression of VM might be a promising strategy to combat this intractable disease.

## Additional files

Additional file 1: MFI and Pos values for 242 cell surface markers. (XLSX 53 kb)

## Data Availability

All data generated or analyzed during this study are included in this article and its additional files.

## Abbreviations

CSC: Cancer stem cell
EGF: Epidermal growth factor
EGFR: Epidermal growth factor receptor
EMT: Epithelial-mesenchymal transition
EPHA2: EPH receptor A2
ER: Estrogen receptor
F-actin: Filamentous actin
FEC: 5-Fluorouracil plus epirubicin plus cyclophosphamide
FGF2: Fibroblast growth factor 2
HER2: Human epidermal growth factor receptor-2
HER2+: HER2-positive
HUVEC: Human umbilical vein endothelial cell
IGF1: Insulin-like growth factor 1
IGF1R: Insulin-like growth factor-1 receptor
IHC: Immunohistochemistry
MAPK: Mitogen-activated protein kinase
MET: Hepatocyte growth factor receptor
NAC: Neoadjuvant chemotherapy
PAS: Periodic acid-Schiff
PI3K: Phosphoinositide-3 kinase
qRT-PCR: Quantitative reverse transcription PCR
SKBR3-TR: Tzm-resistant SKBR3
TGF: Transforming growth factor
Tzm: Trastuzumab
VE-cadherin: Vascular endothelial-cadherin
VEGF: Vascular endothelial growth factor
VM: Vasculogenic mimicry
